# Metagenomic sequencing reveals viral abundance and diversity in mosquitoes from the Shaanxi-Gansu-Ningxia region, China

**DOI:** 10.1371/journal.pntd.0009381

**Published:** 2021-04-26

**Authors:** Xiaozhou He, Qikai Yin, Liwei Zhou, Lei Meng, Weijun Hu, Fan Li, Yang Li, Kun Han, Shaobai Zhang, Shihong Fu, Xiaoshu Zhang, Ji Wang, Songtao Xu, Yi Zhang, Ying He, Maoxing Dong, Xinxin Shen, Zheng Zhang, Kai Nie, Guodong Liang, Xuejun Ma, Huanyu Wang

**Affiliations:** 1 NHC Key Laboratory of Medical Virology and Viral Diseases, National Institute for Viral Disease Control and Prevention, Chinese Center for Disease Control and Prevention, Beijing, People’s Republic of China; 2 Chinese Center for Disease Control and Prevention -Wuhan Institute of Virology, Chinese Academy of Sciences Joint Research Center for Emerging Infectious Diseases and Biosafety, Center for Biosafety Mega-Science, Chinese Academy of Sciences, Wuhan, People’s Republic of China; 3 Department of Arboviruses, NHC Key Laboratory of Biosafety, National Institute for Viral Disease Control and Prevention, State Key Laboratory for Infectious Disease Prevention and Control, Chinese Center for Disease Control and Prevention, Beijing, People’s Republic of China; 4 Ningxia Hui Autonomous Region Center for Disease Control and Prevention, Yinchuan, People’s Republic of China; 5 Gansu Provincial Center for Disease Control and Prevention, Lanzhou, People’s Republic of China; 6 Shaanxi Provincial Center for Disease Control and Prevention, Xi’an, People’s Republic of China; Universita degli Studi di Pavia, ITALY

## Abstract

**Background:**

Mosquitoes host and transmit numerous arthropod-borne viruses (arboviruses) that cause disease in both humans and animals. Effective surveillance of virome profiles in mosquitoes is vital to the prevention and control of mosquito-borne diseases in northwestern China, where epidemics occur frequently.

**Methods:**

Mosquitoes were collected in the Shaanxi-Gansu-Ningxia region (Shaanxi Province, Gansu Province, and Ningxia Hui Autonomous Region) of China from June to August 2019. Morphological methods were used for taxonomic identification of mosquito species. High-throughput sequencing and metagenomic analysis were used to characterize mosquito viromes.

**Results:**

A total of 22,959 mosquitoes were collected, including *Culex pipiens* (45.7%), *Culex tritaeniorhynchus* (40.6%), *Anopheles sinensis* (8.4%), *Aedes* (5.2%), and *Armigeres subalbatus* (0.1%). In total, 3,014,183 (0.95% of clean reads) viral sequences were identified and assigned to 116 viral species (including pathogens such as Japanese encephalitis virus and Getah virus) in 31 viral families, including *Flaviviridae*, *Togaviridae*, *Phasmaviridae*, *Phenuiviridae*, and some unclassified viruses. Mosquitoes collected in July (86 species in 26 families) showed greater viral diversity than those from June and August. *Culex pipiens* (69 species in 25 families) and *Culex tritaeniorhynchus* (73 species in 24 families) carried more viral species than *Anopheles sinensis* (50 species in 19 families) or *Aedes* (38 species in 20 families) mosquitoes.

**Conclusion:**

Viral diversity and abundance were affected by mosquito species and collection time. The present study elucidates the virome compositions of various mosquito species in northwestern China, improving the understanding of virus transmission dynamics for comparison with those of disease outbreaks.

## Author summary

Most traditional viral research methods are limited to screening for a few types of viruses with low throughput. Here, we relied on metagenomic sequencing, which is a broadly non-specific screen for any virus, for monitoring of mosquito-borne viruses in the Shaanxi-Gansu-Ningxia region of China in 2019. The baseline of viral abundance and diversity in local *Culex pipiens*, *Culex tritaeniorhynchus*, *Anopheles sinensis*, and *Aedes* mosquitoes were investigated, with 116 viral species identified in 31 viral families, including *Flaviviridae*, *Togaviridae*, *Phasmaviridae*, *Phenuiviridae*, and some unclassified viruses. This study provides a baseline survey of the viromes of mosquitoes in the study area, highlighting the necessity of strengthened monitoring of mosquito-borne pathogens.

## Introduction

Arthropod-borne viruses (arboviruses) are viruses that are maintained in natural reservoirs and transmitted by blood-sucking arthropods between susceptible vertebrate hosts. More than 500 arboviruses have been identified around the world, of which approximately 130 are capable of causing disease in human and animals [1]. In recent years, researchers in China have successfully isolated 29 viral species belonging to 7 families and 13 genera [2]. Mosquitoes are major vectors that transmit numerous arboviruses to various hosts, causing disease in both humans and animals [3, 4]. For example, the *Aedes aegypti* is the primary vector for Dengue virus, Zika virus, and Chikungunya virus, and *Culex* is a primary vector for Japanese Encephalitis Virus (JEV) and West Nile Virus (WNV) [5, 6]. Mosquitoes thus pose an immense threat to public health due to their role in transmitting viral diseases.

Viromes are structurally and functionally diverse, with differences among habitats in hosts and environments; prior to the development of high-throughput sequencing, little was known about the viromes of invertebrates and vertebrates [7–9]. Viral profiling through metagenomics is a relatively new technique that takes advantage of high-throughput sequencing and is broadly non-specific to identify any viruses present in a given sample [10–12]. Metagenomics methods have provided new insights into the substantial complexity and diversity of viruses carried by mosquitoes, which has significance for dynamic surveillance of pathogens and the response to emerging and remerging infectious diseases [13–15].

The Shaanxi-Gansu-Ningxia geographic region (Shaanxi Province, Gansu Province, and Ningxia Hui Autonomous Region) is located in northwestern China. This area mainly contains hills and gully areas of the Loess Plateau, a typical agricultural and pastoral zone of northern China. In recent years, accompanying the rapid development of the region’s economy and livestock husbandry, frequent population movement, and accompanying climate and environmental changes, the risk of mosquito-borne viral disease transmission in the area has increased [16–19]. The Shaanxi-Gansu-Ningxia region is traditionally a low epidemic area for JEV, but endured a significant outbreak of this disease in 2018, with approximately 833 cases reported [20, 21]. Characterization of the baseline mosquito virome in the Shaanxi-Gansu-Ningxia region is of great importance for assessments of mosquito-borne disease transmission risk and vector competence, and will provide scientific data for the prevention and control of arbovirus disease transmission [22–24]. However, few data have been reported from this region. In the present study, we investigated the virome compositions of various mosquito species in the Shaanxi-Gansu-Ningxia region using a viral metagenomics approach to provide additional insights into the viruses circulating in this region.

## Materials and methods

### Mosquito collection

**Fig 1:**
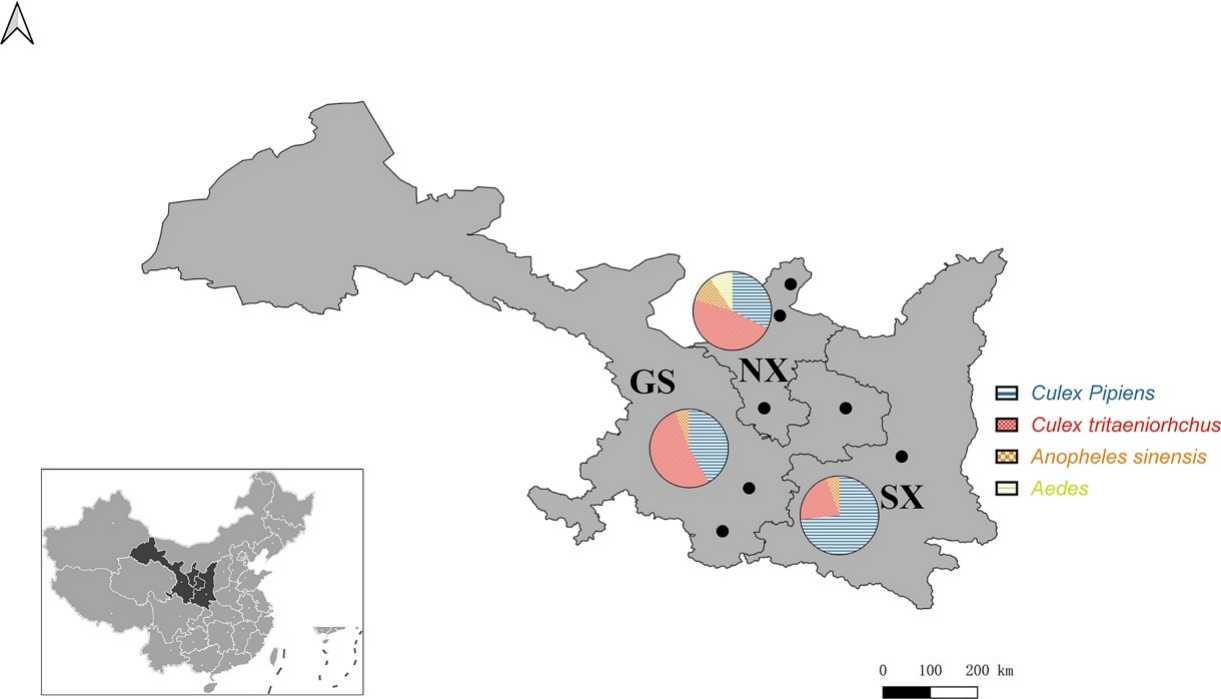
Map of mosquito collection sites. The Shanxi-Gansu-Ningxia region is labeled in light gray and cities where the collection sites were located are indicated with black dots. The pie charts on the map show the composition of mosquito species collected in each province, with each color representing a mosquito species. The location of the Shaanxi-Gansu-Ningxia region is shown in dark gray on the map of China in the lower left of corner (GS: Gansu Province, SX: Shaanxi Province, NX: Ningxia Hui Autonomous Region). The base layer of this modified map originated from National Earth System Science Data Center, National Science & Technology Infrastructure of China (http://www.geodata.cn).

Mosquitoes were collected between June to August of 2019 from seven cities in the Shaanxi-Gansu-Ningxia region (Fig 1). Farms, pigpens, sheep pens, and residential areas were sampled. A trapping lamp was placed outdoors at 18:00–20:00 and retrieved in the early morning of the next day. On an ice bath, male mosquitoes were removed and the species of female mosquitoes were determined based on morphological characteristics. The female mosquitoes were pooled by species, time of collection, and location (30–50 mosquitoes/pool), and then stored at −80°C for subsequent use. The collection sites distribution of mosquito and location of the Shaanxi-Gansu-Ningxia region were presented using ArcGIS (v.10.0; ESRI, Redlands, CA, USA).

### Nucleic acid extraction and high-throughput sequencing

Each mosquito pool was homogenized on an ice bath. Total RNA was extracted with the QIAamp Viral RNA Minikit (QIAGEN) according to the manufacturer’s instructions. cDNA was synthesized using Superscript III reverse transcriptase (ThermoFisher) and random primers, followed by the addition of Klenow enzyme (New England BioLabs) to complete second-strand cDNA synthesis. The REPLI-g Mini Kit (QIAGEN) was used for pre-enrichment via multiple displacement amplification. The NexteraXT DNA Library Preparation Kit (Illumina) was used to construct sequencing libraries. After each library was quantified on the Qubit 4.0 fluorometer (ThermoFisher), sequencing was performed on the Illumina Novaseq (2×150 bp) or MiSeq (2×300 bp) high-throughput sequencing platform. The amount of sequencing data was adjusted according to the number of mosquitoes in each pool to balance the data available for all pools.

### Metagenomics data analysis

The raw paired-end reads obtained from high-throughput sequencing were subjected to quality control using the Trimmomatic (v0.39), BBDUK (v38.79), and Fastp (v0.20.0) tools. Adapters, low-complexity reads, and low-quality bases (with scores less than 15) were removed to generate clean data. Then, the clean reads were aligned to genomes of *Culex quinquefasciatus* (CulPip1.0, RefSeq: GCF_000209185.1), *Anopheles sinensis* (AS2, GenBank: GCA_000441895.2), and *Aedes albopictus* (Aalbo_primary.1, RefSeq: GCF_006496715.1), respectively. All mosquito genomes were available at Genomes–NCBI Datasets (https://www.ncbi.nlm.nih.gov/datasets/genomes/). Unmapped reads were extracted and then paired-end aligned to the NCBI viral whole-genome database (https://www.ncbi.nlm.nih.gov/labs/virus/vssi/) using Bowtie 2 (v2.3.5) in end-to-end mode (with the parameter ‘very-sensitive’). Mapped reads were extracted and duplicate reads were removed using SAMtools (v1.9) to obtain unique virus-related reads. The Blastn alignment function in NCBI-Blast+ (v2.9.0) was used to align the unique reads with the local virus reference genome database in ‘best hit only’ mode. Alignment results with E-values lower than 1e-10 and identity higher than 90% were retained for the next step. Finally, MEGAN6 (v6.20) software was used for metagenomic annotation based on a genomic DNA taxonomic database (Jul2020) with the default lowest common ancestor parameter. Annotations with less than five reads to any reference genome were removed from further analysis. Data visualization was conducted using Graphpad, VIP [25] and TBtools [26]. Sequencing coverage and depth were analyzed with SAMtools. The length coverage percentage of each viral species was calculated using the following formula: total length of read covering the reference / number of bases in the reference. The consensus sequence identity with the reference genome was determined using Blastn.

## Results

### Mosquito collection in the Shaanxi-Gansu-Nanjing region from June to August 2019

In total, 22,959 mosquitoes were collected during the peak mosquito activity period from June to August in the Shaanxi-Gansu-Ningxia region of China (Fig 1 and S1 Table). Among them, *Culex pipiens* (Cx.p) accounted for 45.7% (10,498/22,959) of all mosquitoes, and was the most abundant mosquito species. *Culex tritaeniorhynchus* (Cx.t), *Anopheles sinensis* (An.), *Aedes* (Ae.), and *Armigeres subalbatus* accounted for 40.6% (9322/22,959), 8.4% (1940/22,959), 5.2% (1184/22,959), and 0.1% (15/22,959) of the total mosquitoes, respectively. *Armigeres subalbatus* were removed from further analysis, as the number of mosquitoes was insufficient for library construction.

### Virome profiles of mosquitoes

**Fig 2:**
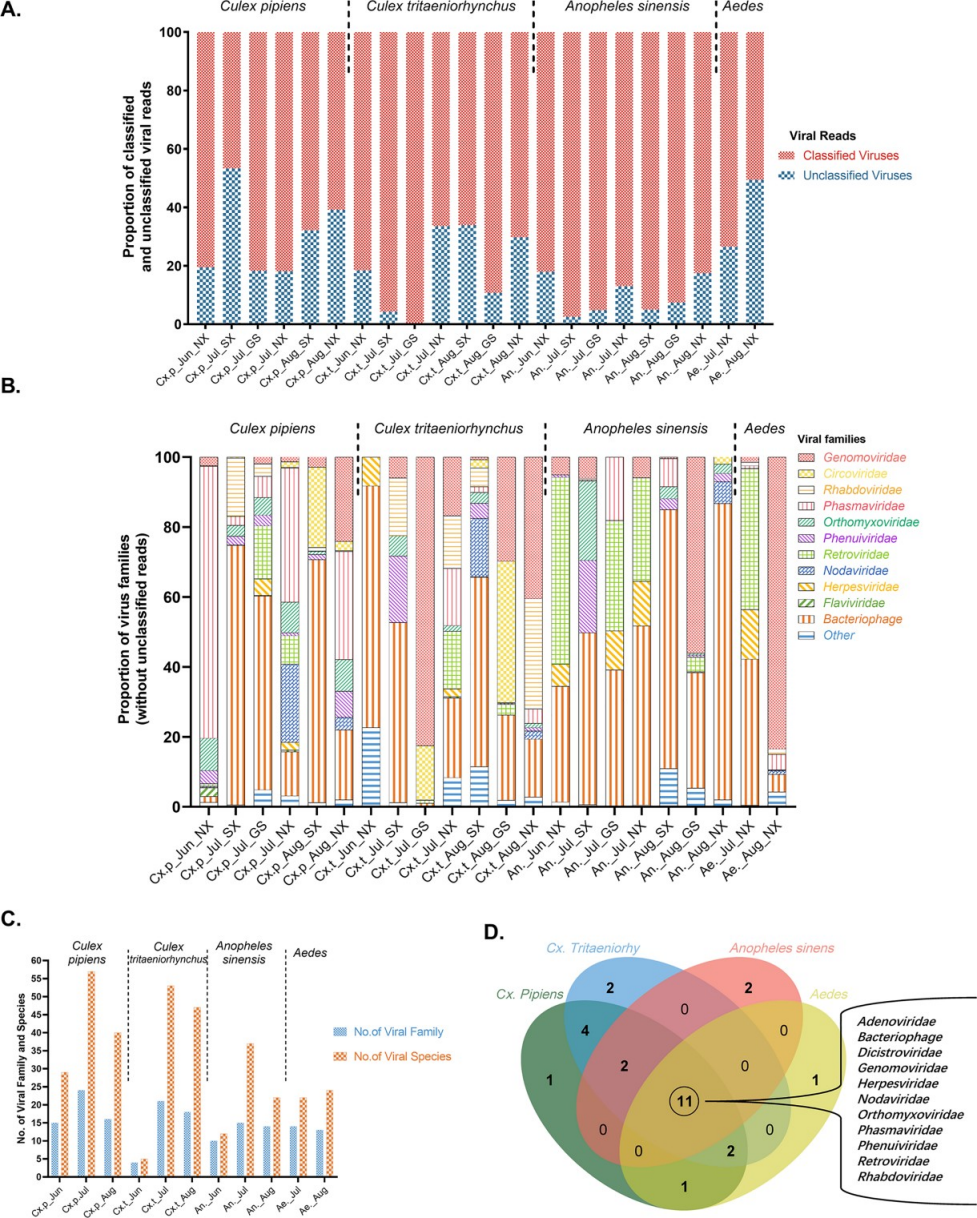
Virome of mosquitoes in the Shaanxi-Gansu-Ningxia region. **A.** Proportions of classified and unclassified viral reads. Each bar represents a mosquito pool, as described in Table 1. **B.** Proportions of viral families among the classified viral reads. Each bar represents a mosquito pool, as described in Table 1. Read percentages lower than 0.1% are grouped as Others. **C.** Number of viral families and species among mosquitoes sorted by mosquito species and collection month. **D.** Venn diagram showing viral families found in the four mosquito groups. The four mosquito groups are presented in separate areas of the diagram. The numbers are representative of viral families found in mosquitos of each genus.

We characterized the viromes of 22,944 mosquitoes in 22 pools, representing four mosquito species from three provinces (Shaanxi, Gansu, and Ningxia) (Table 1). A total of 394,144,760 paired-end reads (55.4 G base pair) were generated through high-throughput sequencing, with an average of about 9.9 M reads per 500 mosquitoes (2.2–23.8 M reads/500 mosquitoes). After quality filtering, 15.8 G bases (77.3 M reads) were removed and 316,821,976 clean reads were retained for the subsequent analysis pipeline. Among those, 3,014,183 reads were identified as viral reads, accounting for 0.95% (3,014,183/316,821,976) of all clean reads, which suggests a rich diversity of viruses harbored by mosquitoes. The proportion of reads aligned to the viral genome database in each pool ranged from 0.01% to 5.96% of clean reads (Table 1). The three pools of Cx.t in July in Gansu, Cx.p in July in Shaanxi, and An. in July in Shaanxi achieved the highest proportions of viral reads, with 5.96%, 3.45%, and 2.99% of clean reads attributed to viruses, respectively. Meanwhile, the proportions of viral reads in all other pools were below 1%. Of the viral reads in each pool, 0.28–53.34% of reads were labeled as unclassified viral sequences, which could not be annotated to families or species (Fig 2A). Unclassified viral reads accounted for 27.74% (836,150/3,014,183) of total viral reads.

**Table 1:** Mosquito collection and sequencing data generation.

Mosquito species	Month	Location	Pool name	No. of mosquitoes	Raw reads	Viral reads	Viral read proportion
*Culex pipiens*	June	Ningxia	Cx.p_Jun_NX	1651	31,927,546	59,303	0.23%
	July	Shaanxi	Cx.p_Jul_SX	2104	47,610,058	1,282,927	3.45%
	July	Gansu	Cx.p_Jul_GS	1647	22,426,930	56,294	0.30%
	July	Ningxia	Cx.p_Jul_NX	2139	40,324,558	142,331	0.43%
	August	Shaanxi	Cx.p_Aug_SX	2750	19,976,476	55,304	0.33%
	August	Ningxia	Cx.p_Aug_NX	207	5,454,292	14,588	0.32%
*Culex tritaeniorhynchus*	June	Ningxia	Cx.t_Jun_NX	27	2,178,600	119	0.01%
	July	Shaanxi	Cx.t_Jul_SX	503	12,653,570	55,281	0.65%
	July	Gansu	Cx.t_Jul_GS	782	15,096,738	769,383	5.96%
	July	Ningxia	Cx.t_Jul_NX	1440	47,547,796	40,818	0.11%
	August	Shaanxi	Cx.t_Aug_SX	900	15,298,094	8379	0.07%
	August	Gansu	Cx.t_Aug_GS	1300	13,032,834	95,124	0.97%
	August	Ningxia	Cx.t_Aug_NX	4370	24,813,632	31,030	0.15%
*Anopheles sinensis*	June	Ningxia	An._Jun_NX	39	7,935,140	1256	0.02%
	July	Shaanxi	An._Jul_SX	64	12,097,926	290,905	2.99%
	July	Gansu	An._Jul_GS	33	15,104,164	6873	0.05%
	July	Ningxia	An._Jul_NX	1034	9,425,030	1367	0.02%
	August	Shaanxi	An._Aug_SX	300	8,799,222	2838	0.04%
	August	Gansu	An._Aug_GS	200	9,246,162	14,362	0.18%
	August	Ningxia	An._Aug_NX	270	8,313,570	366	0.01%
*Aedes*	July	Ningxia	Ae._Jul_NX	374	11,929,360	50,793	0.53%
	August	Ningxia	Ae._Aug_NX	810	12,953,062	34,542	0.32%
	Sum			22,944	394,144,760	3,014,183	

### Viral diversity of classified viruses

The classified viral reads were distributed into 31 viral families and 116 viral species (S2 and S3 Tables). Among them, 90.66% of viral reads belonged to 11 viral families, including *Genomoviridae* (33.11%), *Bacteriophage* (containing six viral families, 34.25%), *Circoviridae* (7.62%), *Rhabdoviridae* (5.7%), *Phasmaviridae* (5.14%), and *Orthomyxoviridae* (4.83%). A further 20 viral families, including plant viruses and viroids, accounted for 9.34% of total classified viral reads (Fig 2B). The highest viral diversity was observed in the pool of Cx.p collected from Gansu in July (57 viral species in 24 viral families) (Fig 2C). Among mosquito species, Cx.p pools (69 species in 25 families) and Cx.t pools (73 species in 24 families) showed greater viral diversity than those of An. (50 species in 19 families) and *Aedes* (38 species in 20 families) (S1 Fig and S4 Table). The pools of Cx.p (57 species in 24 families) and Cx.t (53 species in 21 families) collected in July revealed the highest viral diversity (Fig 2C). Among collection months, we observed that mosquitoes collected in July showed greater viral diversity (86 species in 26 families) than those obtained in June (36 species in 18 families) or August (72 species in 25 families).

Furthermore, at the viral species level, 17 viral species show broad vector ranges encompassing all four mosquito groups, including Culex orthophasmavirus, Wuhan Mosquito Virus 6, Hubei mosquito virus 2, Yongsan picorna-like virus 3, Yongsan picorna-like virus 4, and Culex Hubei-like virus (S2 Fig). Fifty-three viral species showed narrow mosquito vector ranges, of which twenty-five (including JEV) were observed in Cx.t, fourteen in Cx.p, ten in An., and four in *Aedes* (S5 Table). In terms of viral families, eleven families, including *Adenoviridae*, *Herpesviridae*, *Orthomyxoviridae*, *Phasmaviridae*, *Phenuiviridae*, and *Rhabdoviridae*, were identified in all four mosquito groups (Fig 2D). *Anelloviridae* and *Baculoviridae* were only detected in Cx.t, *Poxviridae* and *Xinmoviridae* were only observed in An., and *Togaviridae* were found only in *Aedes*. The families *Luteoviridae*, *Iflaviridae*, and *Flaviviridae* were found in two *Culex* species (S5 Table).

### Viral characterization of selected viruses

**Fig 3:**
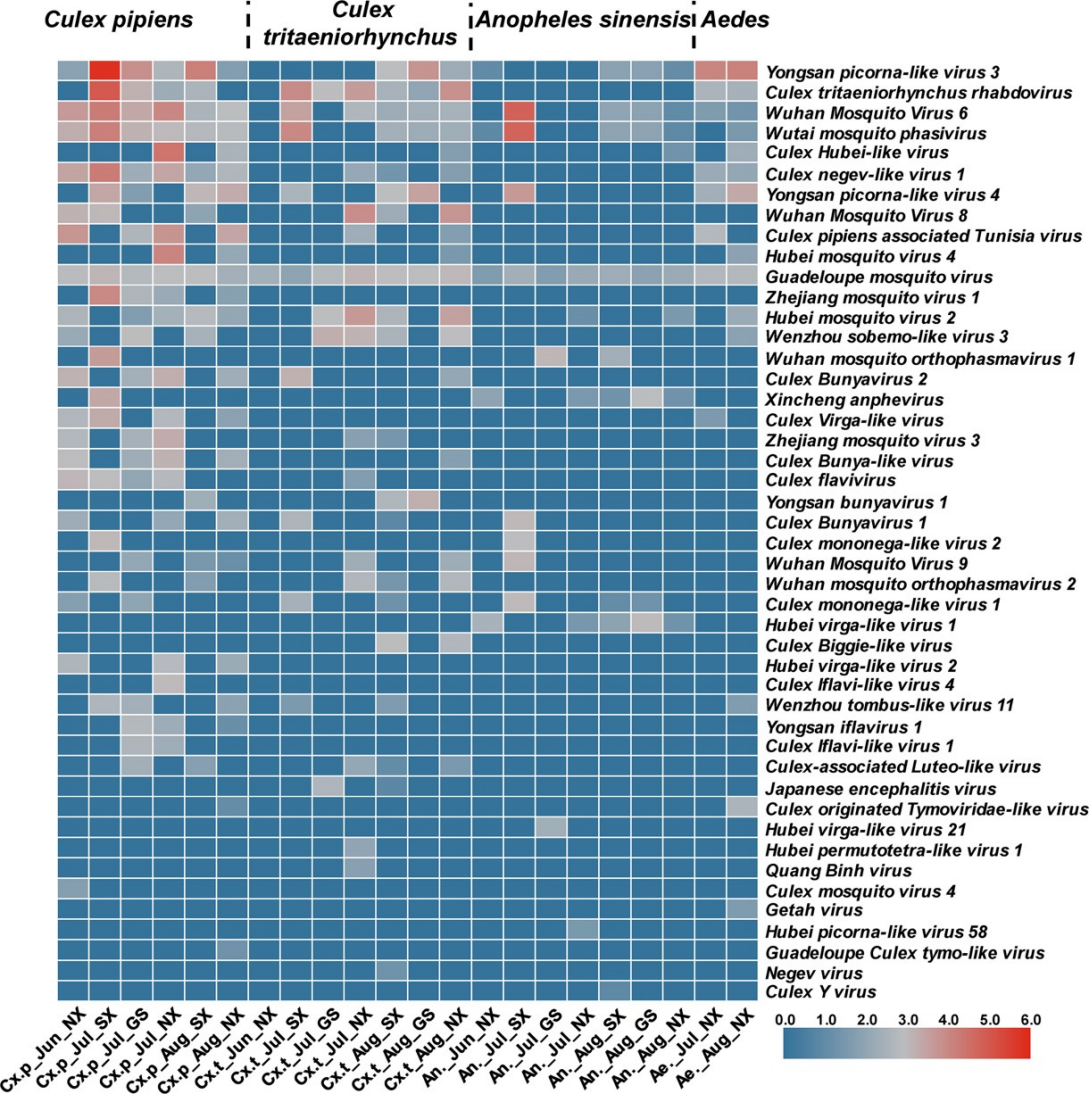
Heatmap showing sequence reads of selected mosquito-borne viruses in each pool. The names of the 46 viral species are listed in the right column. Mosquito species are shown at the top. Pool information is provided in the bottom row. The colors represent the base-10 logarithm of sequence read number, from blue (low read number) to red (high read number).

Forty-six species of mosquito-borne viruses belonging to 13 viral families were discovered in mosquitoes (Fig 3), with a minimum of six and a maximum of 668,502 reads, equivalent to 2.6–100.0% coverage and 1.3–3643.7× of the reference genome. The viral consensus sequences generated here had more than 93.9% nucleotide identity to the corresponding reference genomes (S6 Table). For example, we obtained the near full-length genome of Yongsan picorna-like virus *3* from Cx.p collected in Shaanxi in July, which showed 100% coverage and 98.4% identity with the reference genome (NC040584). Five other viruses also reached greater than 99% coverage and greater than 95% identity to their reference genomes, including Culex Bunyavirus 1 and Wutai mosquito Phasivirus in the family *Phenuiviridae*, as well as Cx.p associated Tunisia virus, Wuhan Mosquito Virus 8, and Hubei mosquito virus 4 in the family *Tymoviridae*.

Through metagenomics, we also identified viral reads assigned to JEV, Culex Flavivirus, and Quang Binh virus belonging to the family *Flaviviridae* genus *Flavivirus* (Table 2). JEV was detected in the pools of Cx.t from Gansu in July (396 reads, 41% coverage, 16.4× depth). The consensus sequences showed 98.1% of nucleotide identity to JEV Genotype-I (HM366552). Culex flavivirus was found in five *Culex* pools (S3 Table). Quang Binh virus was detected in Cx.t from Ningxia in July, with a coverage and identity of 35.2% and 98.1%, respectively, compared to the strain MH827524. Pathogenic flaviviruses such as Yellow fever virus, WNV, and Zika virus were not found. In addition, two species of *Phasmaviridae* and five species of *Phenuiviridae*, which belong to the *Bunyavirales*, were identified in mosquitoes. Getah virus (GETV), belonging to family *Togaviridae* genus *Alphavirus*, was detected in *Aedes* from Ningxia in August with coverage and identity of 19.4% and 98.1% compared to the strain NC_006558, respectively (Table 2).

**Table 2:** Characterization of selected species of mosquito-borne viruses.

Viral family	Viral species	Reference strain	Coverage* (%)	Depth	Per. ident. (%)	Collection month	Collection location	Mosquito species
*Flaviviridae*	Culex flavivirus	NC_008604	98.1	12.8	96.8	Jun	NX	Cx.p
*Flaviviridae*	JEV	HM366552	41.0	16.4	98.1	Jul	GS	Cx.t
*Flaviviridae*	Quang Binh virus	MH827524	35.2	1.9	98.1	Jul	NX	Cx.t
*Iflaviridae*	Yongsan picorna-like virus 3	NC_040584	100.0	3643.7	98.4	Jul	SX	Cx.p
*Phasmaviridae*	Wuhan mosquito orthophasmavirus 1	NC_031307	96.8	62.2	96.6	Jul	SX	Cx.p
*Phasmaviridae*	Wuhan mosquito orthophasmavirus 2	NC_031312	69.4	14.3	97.8	Jul	SX	Cx.p
*Phenuiviridae*	Culex Bunyavirus 1	MH188051	99.3	550.0	95.2	Jul	SX	An.
*Phenuiviridae*	Wutai mosquito phasivirus	NC_031317	99.2	549.8	97.7	Jul	SX	An.
*Phenuiviridae*	Yongsan bunyavirus 1	NC_040759	97.8	29.8	97.5	Aug	GS	Cx.t
*Phenuiviridae*	Culex Bunyavirus 2	MH188052	97.0	29.3	96.3	Jul	NX	Cx.p
*Phenuiviridae*	Culex Bunya-like virus	MH188002	15.0	105.8	97.2	Jul	NX	Cx.p
*Togaviridae*	GETV	NC_006558	19.4	1.8	98.1	Aug	NX	Ae.
*Tymoviridae*	Culex pipiens associated Tunisia virus	NC_040723	99.9	59.5	96.7	Jul	NX	Cx.p
*Tymoviridae*	Wuhan Mosquito Virus 8	NC_028265	99.6	72.3	98.0	Jul	NX	Cx.t
*Tymoviridae*	Hubei mosquito virus 4	NC_032231	99.0	644.9	95.6	Jul	NX	Cx.p

* The coverage (%) indicates the percentage of bases on the reference sequence covered by the reads.

Cx.p (*Culex pipiens*), Cx.t (*Culex tritaeniorhynchus*), An. (*Anopheles sinensis*), SX (Shaanxi Province), GS (Gansu Province), NX (Ningxia Hui Autonomous Region)

## Discussion

Mosquito-borne arboviruses and their associated diseases are a great threat to human health [2, 27]. The life cycle of arboviruses relies on infection of and transmission by arthropod vectors [28]. With changes in climate, urban ecology, high-speed railway and airline travel, and other economic and social activities, the reproduction of mosquito vectors and arboviruses is no longer limited to specific regions [20, 29]. Changes in the population structure of mosquitoes and adaptation by arboviruses to new vectors may increase their geographic distribution. Additionally, some adaptive mutations enhance virus transmissibility by various mosquito species [30]. Thus, some mosquito-borne diseases may become emerging or re-emerging infectious diseases [31]. From the perspective of disease control, tracking the genetic diversity of mutating virus populations with meta-genomic methods will facilitate comparison of virulence traits with available genomic databases, determine disease susceptibility, and enable prediction and prevention of future public health threats [32].

Numerous metagenomic analyses have been conducted in invertebrates such as mosquitoes, spiders, and ticks from many regions of China [9, 22, 33–35], but few efforts have been made to characterize mosquito viromes in northwestern China. In this study, we explored mosquito viromes from seven locations in the Shaanxi-Gansu-Ningxia region using high-throughput sequencing and metagenomics analysis, focusing on identifying the viruses and potential diseases present in mosquitoes. A total of 116 viral species in 31 viral families were observed, including mammalian viruses, arboviruses, and bacteriophages, as well as some virus-related sequences that have not yet been specifically classified. Thus, the spectrum of mosquito-borne viruses is extremely diverse and varies with the time of collection, as well as the type of mosquitoes. Overall, *Culex* mosquitoes play an important role in harboring viruses in the study area, as Cx.p and Cx.t are not only the predominant in mosquitoes in terms of population size but also these species harbor more diverse viruses than other mosquitoes, in accordance with previous reports [5, 6, 36]. *Aedes* is rare in this area, and a small population was observed. In this survey, *Aedes* mosquitoes were collected only in July and August from Ningxia, and they harbored fewer viruses than other mosquito groups (S1 Table). Our results also showed that mosquitoes collected in July harbored greater viral abundance than those from June and August in all *Culex* and *Anopheles* species, but not *Aedes*.

Arboviruses in the families *Flaviviridae* and *Togaviridae*, including JEV, Quang Binh virus, Culex Flavivirus, and GETV, were observed in this study, but no member of *Reoviridae* was found. JEV is a significant human pathogen and GETV causes disease in animals such as pigs and horses. In this study, JEV was found in Cx.t from pigpen environments in Longnan City (Gansu Province in July). The consensus sequences showed close genetic identity (98.1% nt identity) to JEV Genotype-I. As an outbreak of JEV occurred in adults from the Shaanxi-Gansu-Ningxia region in 2018, JEV is of great concern as the main arbovirus disease in the study area. Therefore, monitoring of Cx.t density and tracing the genetic variations of JEV is a vital step in routine surveillance of mosquitoes and mosquito-borne viruses in the study area. In this study, sequence matches to GETV were observed in *Aedes* specimens collected in August from Ningxia Hui Autonomous Region. GETV, a member of the genus *Alphavirus* in the family *Togaviridae*, is an important animal pathogen that causes disease in livestock such as pigs, cattle, and horses [37–39]. GETV was first isolated in Malaysia in 1955, and then spread to Japan, China, South Korea, Mongolia, Russia, and other regions [40]. In China, GETV has been isolated in Yunnan Province, Hebei Province, Shanghai City, Sichuan Province, and Inner Mongolia Autonomous Region [41, 42]. An outbreak was reported at a swine farm in Hunan Province in 2017[43]. *Culex* and *Armigeres* have been reported as the major vectors of GETV [44]. Quang Binh virus was first identified from Cx.t collected in Vietnam in 2002, and has since been reported in southern China, such as Yunnan Province, Guangdong Province, and Shanghai City, but this study was the first report of Quang Binh virus in northwestern China [41, 43, 45]. Routine surveillance of these important pathogens is essential to disease control, and will provide useful information to prevent future outbreaks in this region.

Furthermore, the near-complete genomes of six mosquito-borne viruses were found, including Yongsan picorna-like virus 3, Culex Bunyavirus 1, Culex pipiens associated Tunisia virus, Wuhan Mosquito Virus 8, Hubei mosquito virus 4, and Wutai mosquito phasivirus. These viruses showed high identity with viruses reported in a metagenomic study from China, indicating that they do not exhibit strict geographical or mosquito species restrictions [33, 46]. These viruses can potentially affect the transmission of viruses that are pathogenic to vertebrates, thus representing a potential tool for vector control strategies. However, whether they have any direct or indirect public health risk requires further investigation [47].

A limitation of this study is that the species of *Aedes* mosquitoes were not determined, and all *Aedes* were pooled together without species distinction. Another shortcoming is that this research mainly focused on the viruses present in mosquitoes of the Shaanxi-Gansu-Ningxia region of China, rather than new viral discovery. Our analysis pipeline was based on a known viral reference genome database. Next-generation sequencing may also detect sequences from endogenous viral elements, which have been incorporated into the mosquito genome [23, 24, 48]. To exclude possible endogenous viral elements, each dataset was aligned to the Cx.t, An., and *Aedes albopictus* genomes. However, no mosquito reference genome sequences were available for some mosquito genera; therefore, the effect of this step cannot be evaluated. Moreover, although fewer mosquitoes were collected in June, the differences in sequencing depth and number of mosquitoes among pools were calibrated during the library construction and sequencing processes to support balanced comparison. Mosquitoes collected from seven cities in three provinces were used in this study; in future works, a larger sample size of each mosquito species from each location and coverage of a broader geographic region including more environments are needed. Such information would help to more comprehensively determine the baseline level of mosquito-borne viruses in the study area.

In conclusion, this study provided baseline information on viruses circulating in various mosquito species in the Shaanxi-Gansu-Ningxia region of China, which is of great importance for prediction and prevention of future mosquito-borne disease outbreaks in this region.

## Supporting information

S1 Fig Percentage of viral reads for various mosquito species. A. *Culex pipiens*, B. *Culex tritaeniorhynchus*, C. *Anopheles sinensis*, D. *Aedes*.
(TIF)

S2 Fig Venn diagram showing viral species found in the four mosquito groups. The four mosquito groups are presented in separate areas of the diagram. The numbers are representative of viral families found in mosquitos of each genus.
(TIF)

S1 Table Details of mosquito collection in the Shaanxi-Gansu-Ningxia region from June to August 2019. (DOCX)

S2 Table Viral family matches to reads from each pool. (XLSX)

S3 Table Viral species matches to reads from each pool. (XLSX)

S4 Table Numbers of viral families and species in each pool. (DOCX)

S5 Table Numbers of common viral families and species among mosquito species. (DOCX)

S6 Table Characterization of viral species among mosquito-borne viruses. (XLSX)
