# Supplementary figures and images for: Metagenomic sequencing reveals viral abundance and diversity in mosquitoes from the Shaanxi-Gansu-Ningxia region, China
Source: PLoS Negl Trop Dis. 2021 Apr 26;15(4):e0009381. doi: 10.1371/journal.pntd.0009381 (PMC8101993; doi:10.1371/journal.pntd.0009381)

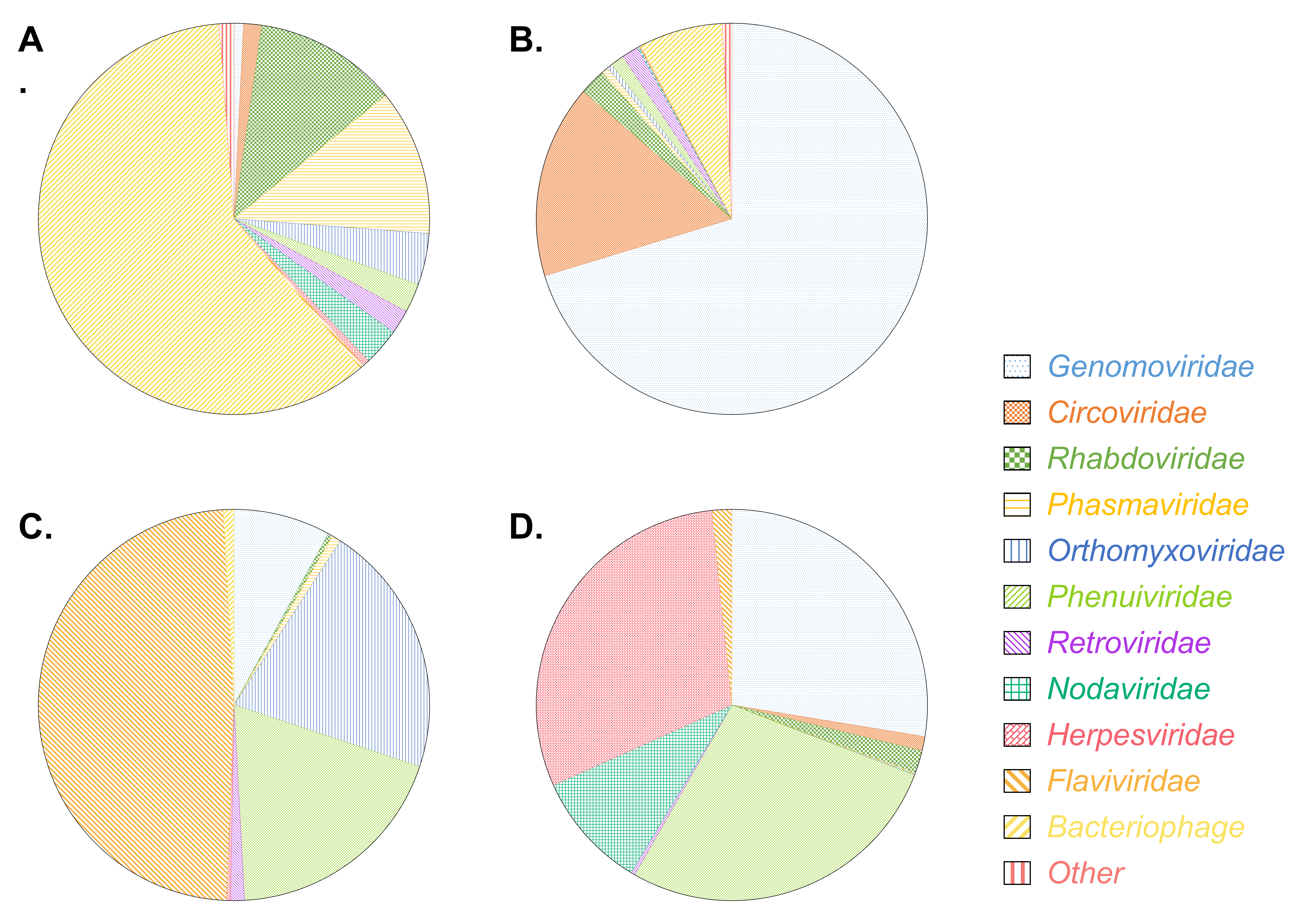

Supplement: S1 Fig — A. Culex pipiens, B. Culex tritaeniorhynchus, C. Anopheles sinensis, D. Aedes. (TIF) [file pntd.0009381.s001.tif]

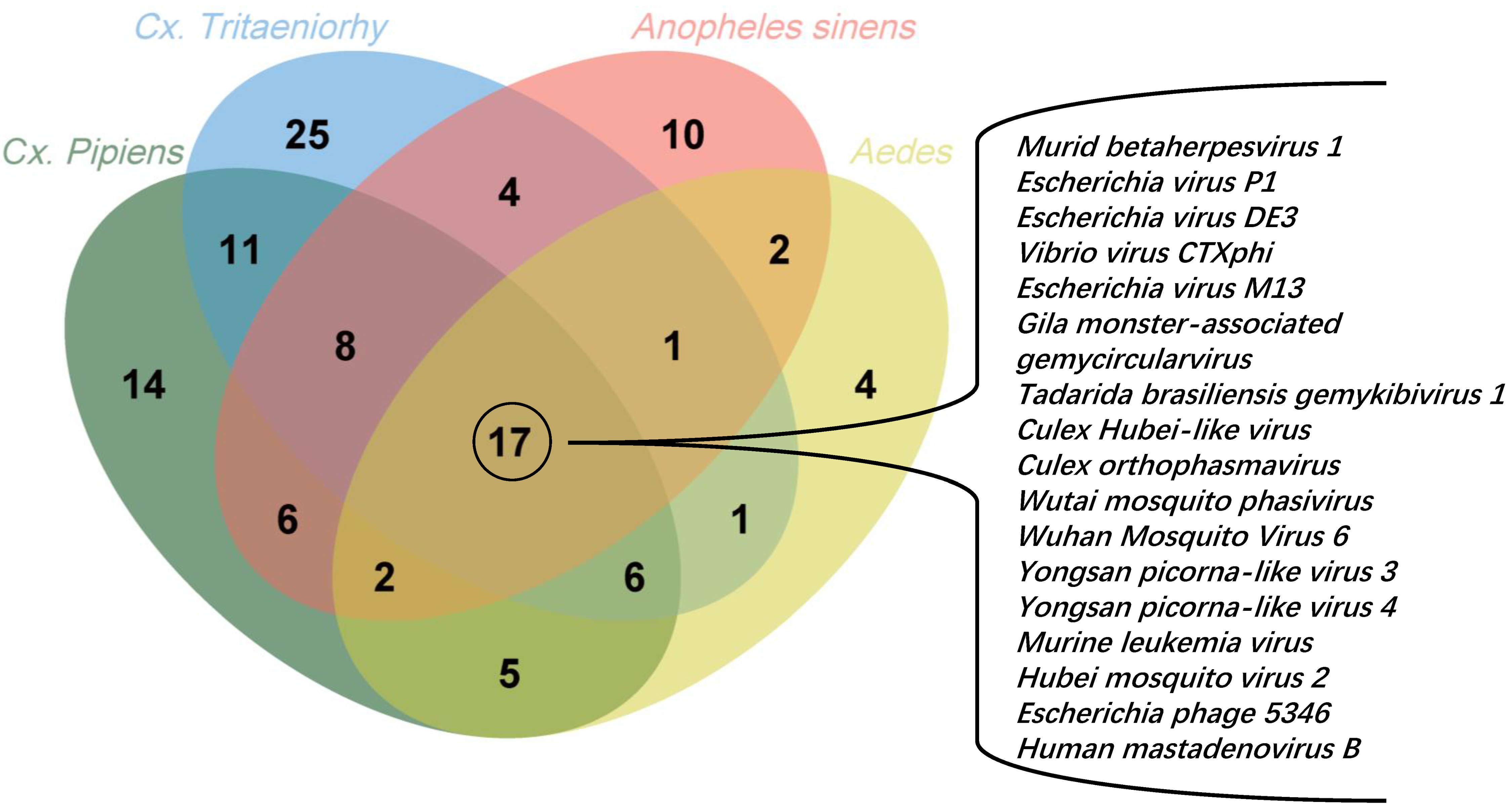

Supplement: S2 Fig — The four mosquito groups are presented in separate areas of the diagram. The numbers are representative of viral families found in mosquitos of each genus. (TIF) [file pntd.0009381.s002.tif]
