# Supplementary material for: Metagenomic sequencing reveals viral abundance and diversity in mosquitoes from the Shaanxi-Gansu-Ningxia region, China
Source: PLoS Negl Trop Dis. 2021 Apr 26;15(4):e0009381. doi: 10.1371/journal.pntd.0009381 (PMC8101993; doi:10.1371/journal.pntd.0009381)
Supplement: S1 Table — (DOCX) [file pntd.0009381.s003.docx]

**S1 Table. Details of mosquito collection in the Shaanxi-Gansu-Ningxia region from June to August 2019**

| Month | Collection location | | | Mosquito species | | | |
| --- | --- | --- | --- | --- | --- | --- | --- |
|  | Province | City | Habitat | *Culex*  *pipiens* | *Culex*  *tritaeniorhynchus* | *Anopheles*  *sinensis* | *Aedes* |
| June | Ningxia | Qingtongxia* | Pigpens Cattle/sheep pens  Dovecote  Courtyard | 1651 | 27 | 39 | - |
| July | Shaanxi | Xianyang | Flower room  Nursery  Sheep pens  Pigpens | 2104 | 503 | 64 | - |
|  | Gansu | Longnan | Residential area Pigpens | 147 | 782 | 33 | - |
|  |  | Tianshui | Park  Residential area | 1500 | - | - | - |
|  | Ningxia | Qingtongxia | - | 1101 | - | - | 204 |
|  |  | Wuzhong | - | 219 | - | - | - |
|  |  | Yinchuan | - | 819 | 1440 | 1034 | 170 |
| August | Shaanxi | Xianyang | Sheep pens | 2750 | 900 | 300 | - |
|  | Gansu | Qingyang | Animal farm  Pigpens | - | 1300 | 200 | - |
|  | Ningxia | Qingtongxia | - | - | 870 | - | - |
|  |  | Shizuishan | - | 80 | 60 | 30 | 570 |
|  |  | Yinchuan | - | 127 | 3440 | 240 | 240 |
| Total | | |  | 10498 | 9322 | 1940 | 1184 |

*county-level city belongs to Wuzhong City
