# Supplementary material for: Metagenomic sequencing reveals viral abundance and diversity in mosquitoes from the Shaanxi-Gansu-Ningxia region, China
Source: PLoS Negl Trop Dis. 2021 Apr 26;15(4):e0009381. doi: 10.1371/journal.pntd.0009381 (PMC8101993; doi:10.1371/journal.pntd.0009381)
Supplement: S4 Table — (DOCX) [file pntd.0009381.s006.docx]

**S4 Table. Numbers of viral families and species in each pool**

**S4.1 Table. Numbers of Viral families and species in each mosquito pool**

**by Mosquito Species**

| Species | No. of Viral Family | No. of Viral Species |
| --- | --- | --- |
| Cx. Pipiens | 25 | 69 |
| Cx. Tritaeniorhynchus | 24 | 73 |
| Anopheles sinensis | 19 | 50 |
| Aedes | 20 | 38 |
| Unique  (Total in all sample) | 31 | 116 |

**S4.2 Table. Numbers of Viral families and species in each mosquito pool**

**by Month**

| Month | No. of Viral Family | No. of Viral Species |
| --- | --- | --- |
| Jun | 18 | 36 |
| Jul | 26 | 86 |
| Aug | 25 | 72 |

**S4.3 Table. Numbers of Viral families and species in each mosquito pool**

**by Species and Month**

| Month | Species | No. of Viral Family | No. of Viral Species |
| --- | --- | --- | --- |
| Jun | Cx.p | 15 | 29 |
| Jun | Cx.t | 4 | 5 |
| Jun | An. | 10 | 12 |
| Jul | Cx.p | 24 | 57 |
| Jul | Cx.t | 21 | 53 |
| Jul | An. | 15 | 37 |
| Jul | Ae. | 14 | 22 |
| Aug | Cx.p | 16 | 40 |
| Aug | Cx.t | 18 | 47 |
| Aug | An. | 14 | 22 |
| Aug | Ae. | 13 | 24 |
