# Supplementary material for: Metagenomic sequencing reveals viral abundance and diversity in mosquitoes from the Shaanxi-Gansu-Ningxia region, China
Source: PLoS Negl Trop Dis. 2021 Apr 26;15(4):e0009381. doi: 10.1371/journal.pntd.0009381 (PMC8101993; doi:10.1371/journal.pntd.0009381)
Supplement: S5 Table — (DOCX) [file pntd.0009381.s007.docx]

**S5 Table. Numbers of common viral families and species among mosquito species**

**S5.1 Table Numbers of Common Viral families among mosquito species**

| Mosquito species | Number | Viral family |
| --- | --- | --- |
| *Cx. Pipiens* | 1 | *Virgaviridae* |
| *Cx. Tritaeniorhy* | 2 | *Anelloviridae*  *Baculoviridae* |
| *Anopheles sinens* | 2 | *Poxviridae*  *Xinmoviridae* |
| *Aedes* | 1 | *Togaviridae* |
| *Cx. Pipiens\|Cx. Tritaeniorhy* | 4 | *Flaviviridae*  *Iflaviridae*  *Luteoviridae*  *Pospiviroidae* |
| *Cx. Pipiens\|Aedes* | 1 | *Tymoviridae* |
| *Cx. Pipiens\|Cx. Tritaeniorhy\|Anopheles sinens* | 2 | *Birnaviridae*  *Circoviridae* |
| *Cx. Pipiens\|Cx. Tritaeniorhy\|Aedes* | 2 | *Narnaviridae*  *Parvoviridae* |
| *Cx. Pipiens\|Cx. Tritaeniorhy\|Anopheles sinens\|Aedes* | 11 | *Adenoviridae*  *Bacteriophage*  *Dicistroviridae*  *Genomoviridae*  *Herpesviridae*  *Nodaviridae*  *Orthomyxoviridae*  *Phasmaviridae*  *Phenuiviridae*  *Retroviridae*  *Rhabdoviridae* |

**S5.2 Table. Numbers of Common Viral species among mosquito species**

| Mosquito species | Number | Viral species |
| --- | --- | --- |
| Cx. Pipiens | 14 | *Acinetobacter phage phiAC-1*  *Dipteran protoambidensovirus 1*  *Pacific flying fox faeces associated gemycircularvirus-8*  *Culex Virga-like virus*  *Guadeloupe Culex tymo-like virus*  *Culex mosquito virus 4*  *Menghai almendravirus*  *Culex Iflavi-like virus 1*  *Culex Iflavi-like virus 4*  *Yongsan iflavirus 1*  *Hubei tombus-like virus 27*  *Hubei tombus-like virus 28*  *Hubei virga-like virus 2*  *Zhejiang mosquito virus 1* |
| Cx. Tritaeniorhy | 25 | *Torque teno sus virus 1a*  *Choristoneura fumiferana granulovirus*  *Klebsiella phage 4 LV-217*  *unclassified Phietavirus*  *Staphylococcus phage SPbeta-like*  *Staphylococcus phage StB2*  *Staphylococcus phage StB2-like*  *Streptococcus phage IPP61*  *Brevidensovirus*  *Porcine parvovirus 2*  *Chicken associated gemycircularvirus 2*  *Gerygone associated gemycircularvirus 2*  *Chicken genomovirus mg7_74*  *Japanese encephalitis virus*  *Quang Binh virus*  *Culex Biggie-like virus*  *Insect inshuvirus*  *Sogatella furcifera honeydew virus*  *Hubei permutotetra-like virus 1*  *Hubei tetragnatha maxillosa virus 5*  *Sanxia permutotetra-like virus 1*  *Wuhan house centipede virus 9*  *Negev virus*  *Chicken virus mg5_2197*  *Thika virus* |
| Anopheles sinens | 10 | *Bovine alphaherpesvirus 5*  *Escherichia phage Rac-SA53*  *Momordica charantia associated gemycircularvirus*  *Culex Y virus*  *Xincheng anphevirus*  *Jaagsiekte sheep retrovirus*  *Hubei picorna-like virus 58*  *Hubei virga-like virus 1*  *Hubei virga-like virus 21*  *Orf virus* |
| Aedes | 4 | *Lepidopteran iteradensovirus 5*  *Bovine associated gemykrogvirus 1*  *Getah virus*  *Rhopalosiphum padi virus* |
| Cx. Pipiens\|Cx. Tritaeniorhy | 11 | *Citrus exocortis viroid*  *Culex flavivirus*  *Culex-associated Luteo-like virus*  *Wuhan mosquito orthophasmavirus 2*  *Culex Bunya-like virus*  *Culex Bunyavirus 2*  *Yongsan bunyavirus 1*  *Zhejiang mosquito virus 3*  *Wuhan Mosquito Virus 8*  *Culex negev-like virus 1*  *Guadeloupe mosquito virus* |
| Cx. Pipiens\|Anopheles sinens | 6 | *Escherichia phage 5465-1*  *Blackfly genomovirus 4*  *Wuhan mosquito orthophasmavirus 1*  *Hubei picorna-like virus 61*  *Culex mononega-like virus 2*  *Campylobacter phage A18a* |
| Cx. Pipiens\|Aedes | 5 | *Escherichia phage 5465-2*  *Enterobacteria phage phi8*  *Hypericum associated gemycircularvirus 1*  *Pteropus associated gemycircularvirus 6*  *Culex originated Tymoviridae-like virus* |
| Cx. Tritaeniorhy\|Anopheles sinens | 4 | *uncultured marine virus*  *Porcine type-C oncovirus*  *Hubei arthropod virus 1*  *Hubei picorna-like virus 66* |
| Cx. Tritaeniorhy\|Aedes | 1 | *Chicken genomovirus mg4_121* |
| Anopheles sinens\|Aedes | 2 | *Human betaherpesvirus 5*  *Escherichia virus T7* |
| Cx. Pipiens\|Cx. Tritaeniorhy\|Anopheles sinens | 8 | *Moraxella phage Mcat16*  *Mosquito associated circovirus 1*  *Finch associated genomovirus 7*  *Wuhan Mosquito Virus 9*  *Culex Bunyavirus 1*  *Aphid lethal paralysis virus*  *Hubei chryso-like virus 1*  *Culex mononega-like virus 1* |
| Cx. Pipiens\|Cx. Tritaeniorhy\|Aedes | 6 | *Aedes japonicus narnavirus 1*  *Culex tritaeniorhynchus rhabdovirus*  *Hubei mosquito virus 4*  *Wenzhou sobemo-like virus 3*  *Wenzhou tombus-like virus 11*  *Culex pipiens associated Tunisia virus* |
| Cx. Pipiens\|Anopheles sinens\|Aedes | 2 | *Macacine betaherpesvirus 3*  *Escherichia virus IME253* |
| Cx. Tritaeniorhy\|Anopheles sinens\|Aedes | 1 | *Porcine feces-associated gemycircularvirus* |
| Cx. Pipiens\|Cx. Tritaeniorhy\|Anopheles sinens\|Aedes | 17 | *Murid betaherpesvirus 1*  *Escherichia virus P1*  *Escherichia virus DE3*  *Vibrio virus CTXphi*  *Escherichia virus M13*  *Gila monster-associated gemycircularvirus*  *Tadarida brasiliensis gemykibivirus 1*  *Culex Hubei-like virus*  *Culex orthophasmavirus*  *Wutai mosquito phasivirus*  *Wuhan Mosquito Virus 6*  *Yongsan picorna-like virus 3*  *Yongsan picorna-like virus 4*  *Murine leukemia virus*  *Hubei mosquito virus 2*  *Escherichia phage 5346*  *Human mastadenovirus B* |
